# A framework for identifying treatment‐covariate interactions in individual participant data network meta‐analysis

**DOI:** 10.1002/jrsm.1300

**Published:** 2018-06-11

**Authors:** S. C. Freeman, D. Fisher, J. F. Tierney, J. R. Carpenter

**Affiliations:** ^1^ MRC Clinical Trials Unit at UCL Aviation House, 90 High Holborn London WC1V 6LJ UK; ^2^ Department of Health Sciences University of Leicester University Road Leicester LE1 7RH UK; ^3^ London School of Hygiene & Tropical Medicine Keppel Street London WC1E 7HT UK

**Keywords:** framework, IPD, network meta‐analysis, treatment‐covariate interaction

## Abstract

**Background:** Stratified medicine seeks to identify patients most likely to respond to treatment. Individual participant data (IPD) network meta‐analysis (NMA) models have greater power than individual trials to identify treatment‐covariate interactions (TCIs). Treatment‐covariate interactions contain “within” and “across” trial interactions, where the across‐trial interaction is more susceptible to confounding and ecological bias.

**Methods:** We considered a network of IPD from 37 trials (5922 patients) for cervical cancer (2394 events), where previous research identified disease stage as a potential interaction covariate. We compare 2 models for NMA with TCIs: (1) 2 effects separating within‐ and across‐trial interactions and (2) a single effect combining within‐ and across‐trial interactions. We argue for a visual assessment of consistency of within‐ and across‐trial interactions and consider more detailed aspects of interaction modelling, eg, common vs trial‐specific effects of the covariate. This leads us to propose a practical framework for IPD NMA with TCIs.

**Results:** Following our framework, we found no evidence in the cervical cancer network for a treatment‐stage interaction on the basis of the within‐trial interaction. The NMA provided additional power for an across‐trial interaction over and above the pairwise evidence. Following our proposed framework, we found that the within‐ and across‐trial interactions should not be combined.

**Conclusion:** Across‐trial interactions are susceptible to confounding and ecological bias. It is important to separate the sources of evidence to check their consistency and identify which sources of evidence are driving the conclusion. Our framework provides practical guidance for researchers, reducing the risk of unduly optimistic interpretation of TCIs.

## 1 BACKGROUND

### 1.1 Introduction

Stratified medicine seeks to identify patients most likely to respond to treatment. However, individual trials are rarely powered to detect interactions between treatment effects and participant characteristics. Meta‐analysis (MA) models potentially have greater power to identify such treatment‐covariate interactions (TCIs), particularly when individual participant data (IPD) are available. One of the big advantages of IPD MA over aggregate data  MA is the greater power that it affords for investigating treatment and patient‐level covariate interactions.^1^, ^2^, ^3^, ^4^ To explore how the treatment effect may vary in relation to a patient‐level covariate, a TCI can be fitted.^5^, ^6^ The power to detect a TCI will depend on the distribution of the covariate within each study.^7^

Pairwise IPD MA of a TCI often results in 2 sources of information—so‐called within‐trial (at the individual patient level) and across‐trial(at the trial level) interactions—where the across‐trial interaction is particularly susceptible to confounding and ecological bias as it is based on observational associations.^1^, ^2^, ^6^, ^7^, ^8^, ^9^, ^10^ The same is true of IPD network MA(NMA); therefore, it is important that the within‐ and across‐trial interaction estimates are reviewed separately, before deciding whether to combine them. We reiterate that confounding from unmeasured covariates (eg, differences in baseline disease risk) can affect both within‐ and across‐trial interactions.^11^ However, within‐trial effects are less susceptible because of the protection provided by the trial randomisation. Ecological bias arises if across‐trial information, which uses trial average covariate values, is used to draw conclusions about individuals.^7^, ^12^, ^13^, ^14^ However, if interest lies in the population level effect and it is interpreted correctly as a population effect (and not an individual effect), then (by definition) there cannot be ecological bias. In some instances, it may be the case that the across‐trial interaction (eg, the mean effect for a patient aged 50) truly differs from the within‐trial interaction (eg, the effect for an individual patient aged 50).^7^ Separating out within‐ and across‐trial interactions may change the conclusions drawn from combining within‐ and across‐trial interactions. For example, an IPD MA comparing 2 antiepileptic drugs as monotherapy for controlling seizures originally combined the within‐ and across‐trial interactions and identified an interaction between treatment and age.^15^ However, a recent reanalysis separating out the within‐ and across‐trial interactions no longer indicated an interaction between treatment and age on the basis of the within‐trial interaction only.^6^

Individual participant data MA models separating out within‐ and across‐trial interactions were first developed for continuous outcomes^8^ and later applied to time‐to‐event outcomes.^16^ In the IPD NMA setting, models separating out within‐ and across‐trial interactions have been proposed for dichotomous outcomes^5^ and time‐to‐event outcomes using the Cox regression model.^6^ In this paper, we show how to separate out the within‐ and across‐trial interactions in the IPD NMA setting for time‐to‐event outcomes using the Royston‐Parmar model.

A key assumption of NMA is consistency between the direct and  indirect evidence.^17^, ^18^ The inclusion of TCIs in a NMA model offers one of many ways for exploring and understanding inconsistency.^5^, ^19^, ^20^, ^21^ With the presence of TCIs in a NMA model, the consistency assumption may be violated if one or more of the true treatment effects is modified by a covariate and included trials differ with respect to the covariate.^5^ Furthermore, when we include TCIs in a NMA model, we assume that the treatment effects estimated at the covariate value of 0 are consistent and that the regression coefficients for the TCI parameters are also consistent.^22^ Therefore, it is important to assess the consistency assumption either at each level of the covariate (for categorical covariates) or across a range of values (for continuous covariates).^22^

There are 3 different ways to model TCI effects: common, independent, or exchangeable.^19^ Common effects assume that the regression coefficients are the same for all TCIs so that the TCI effect is the same for each treatment compared with the control. Independent effects assume that all TCIs are different for each treatment versus the control so that a separate regression coefficient for each TCI is included in the model. Exchangeable effects assume that all TCIs are different from each other but similar enough that they can be sampled from a common distribution. For IPD MA, and as we show by extension in IPD NMA, there are 3 possible ways of analysing TCIs: using the across‐trial interaction only, using the within‐trial interaction only, and combining the two.^23^ We now describe 3 approaches for IPD MA with TCI before considering the NMA setting.

In one commonly used approach for interactions in MA (which can be shown to combine within‐ and across‐trial interactions), specifically for categorical covariates, the treatment effect is calculated within each trial for each level of the covariate. The treatment effects for each level of the covariate are combined across all trials, using standard MA techniques, resulting in an overall effect for each level of the covariate, which are then compared with each other.^8^, ^16^, ^23^ Any trials where all patients have the same covariate value will not contribute to the within‐trial interaction but can contribute to the across‐trial interaction. This is a common approach used in IPD MA. However, the within‐trial interaction can be exaggerated or masked by the across‐trial interaction, which is at risk of ecological bias. Therefore, this approach is also at risk of ecological bias.^8^, ^16^, ^23^

An analysis using the across‐trial interaction only considers how the treatment effect varies across trials in relation to the trial mean value of the covariate and fails to use the patient‐level information.^24^ This requires the assumption of no unmeasured confounding between the outcome and the covariate, and that there is no ecological bias.^8^, ^9^, ^23^ Unfortunately, it is typically not possible to identify such confounders as baseline data often vary across trials. Therefore, it is often not possible to test whether the inclusion of the across‐trial interaction will induce bias.

An analysis using the within‐trial interaction only more closely parallels the underlying principles of MA. Estimates of the TCI effect are calculated within each trial and then pooled together using MA methods.^7^, ^8^, ^16^ Any trials where all patients have the same covariate value will not contribute to this analysis as they do not provide any within‐trial interactions.^7^, ^8^, ^23^ Recommendations on the presentation and analysis of TCIs using this approach are proposed by Fisher et al.^23^ A key aim of this paper is to show how these recommendations can be brought to bear in NMA.

In the pairwise MA case, it is clear that within‐trial interactions are the most clinically relevant estimates, as they are free from ecological bias.^1^, ^5^, ^6^, ^8^, ^23^ In the NMA case, more research is needed to explore how the consistency assumptions of the network applied to within‐trial interactions (as in our model) can help to improve their precision. Nevertheless, the framework described here explicitly separates the within‐ and across‐trial interactions throughout the network  and hence guarantees that an unbiased estimate of the within‐trial interaction is obtained. The methods proposed here are applicable to both continuous and categorical covariates. Specifically, in this paper, we illustrate how the within‐ and across‐trial interactions can be separated for time‐to‐event outcomes modelled using the IPD Royston‐Parmar NMA model, and we propose a framework for conducting NMA with TCIs, showing how to fit models that separate out the within‐ and across‐trial interactions. We then illustrate our framework by applying it to a cervical cancer network.

### 1.2 Why do we need a framework?

Fitting a NMA with TCIs is often a more complex process than researchers anticipate. There are a number of additional important decisions that need to be taken, beyond those that need to be considered in a MA. These include the parameterisation and consistency of covariate and interaction effects. An added complication, frequently encountered in practice, is how to handle missing patient‐level covariate data.

When it comes to reporting a NMA with TCIs, the Preferred Reporting Items for Systematic Reviews and Meta‐Analyses based on IPD guidelines recommend prespecification of whether the across‐trial interaction is to be combined with the within‐trial interaction.^4^, ^25^ Therefore, it is important that authors are aware of the potential implications of combining within‐ and across‐trial interactions. By proposing a framework for conducting NMA with TCIs, we aim to equip researchers with the knowledge and tools for successfully fitting an appropriate NMA with TCIs.

In Section 2, we discuss issues to consider before conducting NMA with TCIs, outline a 9‐step framework for 1‐stage IPD NMA with TCIs, provide guidance on implementing the framework, and introduce a cervical cancer dataset. In Section 3, we present the results of applying the framework to the cervical cancer network. In Section 4, we discuss the framework before drawing some conclusions in Section 5.

## 2 METHODS

### 2.1 Issues to consider

#### 2.1.1 Preliminary analysis

A NMA often starts with a systematic review being conducted to identify all treatments and trials to be considered in the network. As part of the review and in discussion with appropriate clinicians, discussion of any covariates that could be included in a NMA with TCIs should take place before any models are fitted. Such models require a number of considerations, and preliminary analysis of the data can help inform the decision of which model to fit.

#### 2.1.2 Common main effect vs trial‐level main effect of covariate

A patient‐level covariate can be fitted as a common effect or a trial‐level effect.^26^ A common effect pools the effect of the covariate across all trials. A fixed‐trial effect results in a separate estimate of the effect of the covariate for each trial and does not provide an overall effect for the covariate. A random trial effect of a covariate allows the effect of the covariate to differ in each trial assuming that the coefficients for each trial come from a common (typically normal) distribution.

We encourage the use of a trial‐level effect, either fixed or random. If a common effect of a covariate is used when a trial‐level effect would be more appropriate, this can result in a poorly fitting model, which could affect convergence, suppress the differences between trials, and affect the treatment effect estimates. Assuming a common effect of a covariate is generally not appropriate when the distribution of the covariate varies between trials or in a network where trials vary in size, because it is known that smaller studies can give more extreme parameter estimates.^27^ A hypothesis test to check the effect of the covariate in each trial should be conducted before assuming a common effect, as this choice is likely to critically impact the estimate of the TCI.

#### 2.1.3 Parameterisation of within‐ and across‐trial interactions

A TCI can be included in MA models in 2 ways: firstly, as a single effect that combines within‐ and across‐trial interactions and secondly, as 2 effects that separate out the within‐ and across‐trial interactions.^8^, ^16^ We now describe how to do this in the NMA setting using the Royston‐Parmar model for time‐to‐event data.

Consider the 1‐step fixed treatment effect (FTE) Royston‐Parmar NMA model for a network of *q*+1 treatments.^28^By including a fixed‐trial effect of a patient‐level covariate *z*_*ij*_, the log cumulative hazard for patient *i* from trial *j* can be modelled as  

(1) $$\ln\{H_{j}(t|x_{ij})\}=s_{j}\left(\ln(t)\right)+\beta_{1}{\text{trt1}}_{ij}+\;\text{⋯}+\beta_{q}\text{trt}q{}_{ij}+\alpha_{j}z_{ij},\;$$

where $s_{j}\left(\ln(t)\right)$ is the restricted cubic spline for trial *j*, trt1_*ij*_,…,trt*q*_*ij*_ are the treatment indicators with corresponding coefficients *β*_1_,…,*β*_*q*_, and *α*_*j*_ is the effect of the patient‐level covariate *z*_*ij*_ for trial *j*.

Adding a common TCI to (1), which separates the within‐ and across‐trial interactions, results in 

(2) $$\begin{array}{ccc} \ln\{H_{j}(t|x_{ij})\} & =s_{j}\left(\ln(t)\right)+\beta_{1}{\text{trt1}}_{ij}+\;\text{⋯}\; & \\ & \;+\beta_{q}\;\text{trt}q{}_{ij}+\alpha_{j}(z_{ij}-{\bar{z}}_{j})\\ & \;+\delta_{A1}{\text{trt1}}_{ij}(z_{ij}-{\bar{z}}_{j})+\;\text{⋯}\\ & \;+\delta_{Aq}\;\text{trt}q{}_{ij}(z_{ij}-{\bar{z}}_{j})\\ & \;+\delta_{B1}{\text{trt1}}_{ij}{\bar{z}}_{j}+\;\text{⋯}+\delta_{Bq}\text{trt}q{}_{ij}{\bar{z}}_{j}, \end{array}$$

where the covariate *z*_*ij*_ is fitted as a fixed trial‐level effect with coefficient *α*_*j*_ for trial *j* and ${\bar{z}}_{j}$ is the mean value of *z*_*ij*_ for trial *j*. The within‐trial interaction is represented by the *δ*_*A*1_,…,*δ*_*Aq*_ parameters while the across‐trial interaction is represented by the *δ*_*B*1_,…,*δ*_*Bq*_ parameters. The difference *δ*_*Bk*_−*δ*_*Ak*_ quantifies the amount of ecological bias for interaction *k*.^16^

Adding a common TCI to (1), which combines the within‐ and across‐trial interactions, results in 

(3) $$\begin{array}{ccc} \ln\{H_{j}(t|x_{ij})\} & =s_{j}\left(\ln(t)\right)+\beta_{1}{\text{trt1}}_{ij}+\;\text{⋯}+\beta_{q}\text{trt}q{}_{ij}+\alpha_{j}z_{ij} & \\ & \;+\delta_{1}{\text{trt1}}_{ij}z_{ij}+\;\text{⋯}+\delta_{q}\text{trt}q{}_{ij}z_{ij}, \end{array}$$

where the covariate *z*_*ij*_ is fitted as a fixed trial‐level effect with coefficient *α*_*j*_ for trial *j* and *δ*_1_,…,*δ*_*q*_ are the coefficients of the TCI effects.

In practice, these models can also be fitted with random treatment effects (RTEs), random trial‐level TCIs, and random trial‐level effect of the covariate (Appendix S1).

#### 2.1.4 Missing covariate data

A NMA can be conducted in both the frequentist and Bayesian frameworks.^29^, ^30^ One of the advantages of conducting NMA within a Bayesian framework, and in particular using WinBUGS, is that missing covariate data can be naturally handled in WinBUGS. Missing covariate data can be accommodated within the NMA model by including a distribution for the covariate with missing values. This allows 2 things to happen: Missing covariate values are imputed, which allows a patient to be included in the NMA model, and this in turn increases the precision of the treatment effects, which themselves inform the imputation of the missing values.^31^ If we wish to perform a frequentist analysis, the most straightforward way to handle missing covariate values is by multiple imputation; however, this is not a straightforward application of multiple imputation, because the imputation needs to be done in a way that is consistent with the NMA model. The R‐package jomo^63^ has the flexibility to handle this, but we do not pursue this further.

### 2.2 Nine‐step framework for 1‐stage IPD NMA with treatment‐covariate interactions

The aim of this framework is to provide guidance on the steps that need to be considered before a NMA with TCIs can be fitted, so that the analysis is conducted systematically and appropriately. This framework concentrates specifically on building NMA with TCI models. Therefore, the framework assumes that the usual MA activities such as protocol writing, defining inclusion/exclusion criteria, and determining whether trials are similar enough for inclusion in a MA have already been conducted. Further, we assume that the network is connected and any covariates for inclusion in the NMA models have been identified through discussion with clinicians. As usual, when interpreting the results, a range of possible causes of heterogeneity (eg, baseline differences, design, treatment doses, delivery, and escape therapies) must be kept in mind. Steps 1 to 4 are applicable for any NMA whether covariates are considered for inclusion or not. From step 5 onwards, the framework specifically considers the inclusion of covariates and TCIs. This framework has been developed to be applicable to a range of outcomes (eg, binary, continuous, and time‐to‐event). As is common, we work on the log‐odds or log hazard scale. However, the principles underlying our proposed framework will apply to other settings, but the technical details may vary. The framework may need to be tweaked to take into account additional issues arising in specific settings.

1. Assess all pairwise treatment comparisons for evidence of heterogeneity. 
  (a) If heterogeneity is present, explore the baseline characteristics of all trials. Can the heterogeneity be explained by differences in baseline characteristics across trials?
    (i) If yes, all important covariates should be considered going forwards. 
    (ii) If no, it could be unsuitable to combine the pairwise comparison in a NMA. 
2. Identify a reference treatment across the network and determine which treatment contrasts will be parameterised in the model (all other treatment contrasts should be obtained through consistency equations). 
3. Fit the NMA model without covariates taking into account any heterogeneity identified in step 1 (eg, by using a random‐effects model). 
4. Assess the network for evidence of inconsistency. 
5. Investigate patterns of missing data for the covariate of interest. 
6. Consider modelling assumptions for including the covariate in the NMA model (FTE or RTE ? common, fixed‐trial, or random‐trial effect of covariate?)
7. Fit NMA model including covariate and assess model results. 
8. Fit NMA model including TCIs with within‐ and across‐trial interactions separated and assess agreement between the within‐ and across‐trial interactions. 
9. Fit NMA model including TCIs with within‐ and across‐trial interactions combined, if appropriate. 

Note that when there are missing covariate data, it is often practically and computationally easier to fit step 9 before step 8. More details can be found in Section 2.3.

### 2.3 Guidance on implementing the framework

Step 1: Before a NMA model is fitted, all pairwise treatment comparisons in the network should be explored for evidence of heterogeneity. Heterogeneity can be assessed through the *I* ^2^, *τ* ^2^, and Cochran *Q* statistics.^32^, ^33^, ^34^ If heterogeneity is present, explore the baseline characteristics of all trials. If one trial, or a subgroup of trials, is found to be causing the heterogeneity, then exploring the baseline characteristics can identify what is different about this trial, or trials, and the impact this might have on the treatment effect. The identification of heterogeneity, at this stage, in one or more pairwise comparisons can determine whether FTE or RTE models are used in step 4.^35^ If the source of heterogeneity cannot be identified or accounted for, then either RTE will need to be used or it will be unsuitable to use this comparison in a NMA, particularly if removing the pairwise comparison exhibiting heterogeneity means that a FTE model can be used. Any covariates identified, during this step, as potentially causing heterogeneity should be considered in steps 6 to 9.
Step 2: The previous standard of care, the largest treatment node, and the treatment connected to the greatest number of other treatments are the most appropriate choices for the reference treatment within the network. The treatment parameterisation of the network should satisfy the consistency equations.^36^ The number of treatment parameters should be one less than the number of treatments in the network. The network diagram can help inform which treatment parameters should be directly estimated in the NMA model and which will be calculated as contrasts through the consistency equations. Network diagrams can be created in Stata^37^ using the *networkplot* command.^38^
Step 3: A NMA model can be fitted using both FTE and RTE and monitoring the deviance information criteria (DIC). If heterogeneity was present in step 2, the RTE model should be used as this increases the variability around the point estimate to reflect the heterogeneity. The RTE model gives more weight to smaller studies than the FTE model does. Therefore, a difference in the treatment effect estimates between the FTE and RTE models can indicate publication bias and small study effects.^27^The DIC is a measure of model fit, which penalises model complexity—smaller values are better. The DIC can be used to compare models, although small differences (ie, <5) should not be overinterpreted and simpler models should be chosen where they can be.^39^ In addition, in some cases, total residual deviance can be used to assess goodness of fit; see Dias et al^18^ for details.
Step 4: The network should be assessed for evidence of inconsistency.^5^, ^21^, ^40^, ^41^, ^42^, ^43^, ^44^ To visualise this, it is useful to present the model results as a forest plot with the network, direct, and indirect evidence separated out. There are many approaches to assessing inconsistency (eg, node‐splitting,^45^, ^46^, ^47^ inconsistency models such as the design‐by‐treatment interaction model,^48^, ^49^ random inconsistency effects,^50^, ^51^, ^52^ factorial analysis of variance,^53^ generalised linear mixed models,^54^, ^55^ and the 2‐stage approach^56^. We recommend consulting review papers such as Donegan et al^20^ and Efthimiou et al,^57^ which describe and compare different methods for assessing consistency to help select the most appropriate method for the network at hand.
  We use the inconsistency parameter approach of Lu and Ades^42^ in which an inconsistency parameter is fitted for each treatment loop and the model is refitted including the additional parameters. This approach complements the assessment of heterogeneity from step 1 and follows the approach outlined in Freeman and Carpenter.^28^ Here, an inconsistency parameter is initially added to the FTE model before considering the RTE model and exploring whether the conclusions are sensitive to the inclusion of the inconsistency parameter. If inconsistency is present in the network, then an inconsistency parameter can be used in all further models. Treatment loops with inconsistency parameters are reduced to the direct evidence only and therefore do not contribute to the across‐trial interaction in the network. Furthermore, if inconsistency is present, the cause of the underlying inconsistency/heterogeneity must be resolved before the results are used for clinical inference.^22^, ^28^
Step 5: Consider the distribution of the covariate of interest in each trial. Are there any trials where some patients have missing covariate data? Are there any trials where all patients have missing covariate data? Is the covariate continuous or categorical? Can a linear effect between the groups of an ordered categorical covariate be assumed? What is the reference value of the covariate? In WinBUGS,^58^ as in all Bayesian modelling software, missing covariate data can be imputed once the marginal distribution of the covariate is specified.
Step 6: Covariates can be included as common effects, fixed trial‐level effects, or random trial‐level effects. The DIC can be used to determine which of these assumptions is most appropriate. However, assuming a common effect of a covariate is only likely to be appropriate if the distribution of the covariate is the same in every trial in the network. The choice of FTE or RTE should be informed by previous steps such as the presence of heterogeneity from step 2 or the DIC from step 4.
Step 7: Fit the NMA models including the patient‐level covariate and assess the results. This can help inform the decision of which NMA model with TCI to fit.  TCIs can be fitted as  common effects, fixed trial‐level effects, or random trial‐level effects. A common effect assumes that the TCI has the same effect in all trials. A random trial‐level effect allows the effect of the TCI to differ in each trial but assumes that the coefficients for each trial come from a common (typically normal) distribution. The choice of assumption for TCIs can be informed by the distribution of the covariate within and across trials.
Step 8 and 9: The framework recommends that the within‐ and across‐trial interactions are considered separately at first and then combined if it is appropriate to do so. However, the mean covariate value from each trial is needed to separate out the within‐ and across‐trial interactions and to calculate this missing covariate data needs to be imputed. Although it is possible, in principle, to impute the missing covariate data, calculate the mean covariate value, and fit the NMA model separating within‐ and across‐trial interactions in one step, in practice, software is unlikely to do this. Therefore, it is more practical and computationally easier to fit the model combining within‐ and across‐trial interactions first and monitor the mean value of the covariate in any trials with missing covariate data before using these values to fit the model separating the within‐ and across‐trial interactions. For trials with only some missing covariate data, the weighted average of the mean observed value and mean imputed value of the covariate can be used as the trial mean value. In trials where all patients have missing covariate values, the mean covariate value from the imputed values can be used as the trial mean value. A sensitivity analysis in which patients with missing covariate data are excluded can be conducted to check that the imputation of the missing covariate data has been handled correctly.
  A visual assessment of the agreement between the within‐ and across‐trial interactions can be made by plotting the parameter estimates for the TCIs. Log hazard ratios (LogHR) along with 95% credible intervals (CrI) can be presented in tables. If TCIs are present, the treatment effect parameters on their own do not have a useful interpretation. Treatment effects should be presented separately for each level of the covariate. Consistency can then be checked for each level of the covariate following methods described by Donegan et al.^22^ Graphs ranking the treatments for each level of the covariate can be used as a visual aid for determining the most effective treatment for each level of the covariate.^38^, ^59^

### 2.4 Example

**Figure 1:**
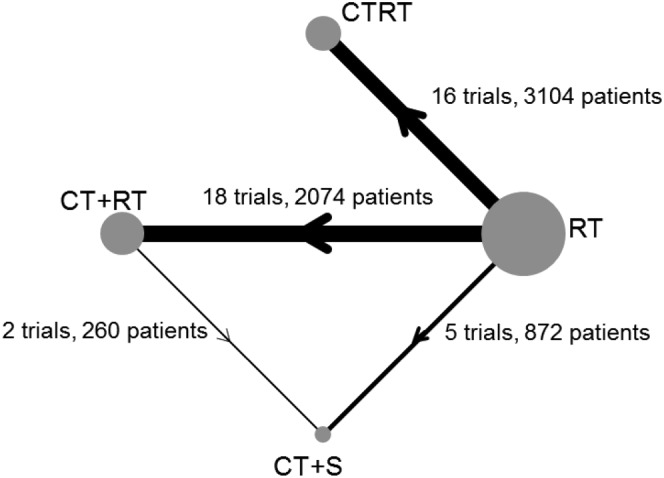
Cervical cancer network diagram. Node size is proportional to the number of patients randomised to each treatment, and line thickness is proportional to the number of studies involved in each direct comparison. Note that this network diagram includes the main set of 13 RT vs CTRT trials only (which in this paper is analysed as 16 trials owing to the splitting of 3 four‐arm trials each into 2 unconfounded comparisons of RT vs CTRT), and the number of patients for each treatment arm does not add up to the total number of patients included in the network, because multiarm patients are counted twice. There are a total of 37 trials in this network. However, in the figure, the 2 multiarm trials are counted 3 times each as they are included in the number of trials for each pairwise comparison. CT+RT indicates neoadjuvant chemotherapy plus radiotherapy; CT+S, neoadjuvant chemotherapy plus surgery; CTRT, chemoradiation; RT, radiotherapy

Our example comes from 3 MAs of randomised controlled trials (RCTs) in cervical cancer performed by 2 international collaborations.^60^, ^61^ The 3 MAs considered 4 different treatments: radiotherapy (RT), chemoradiation (CTRT), neoadjuvant chemotherapy plus radiotherapy (CT+RT), and neoadjuvant chemotherapy plus surgery (CT+S) (Figure 1).

The RT vs CTRT comparison included a total of 18 RCTs and 4818 patients. In the original publication, 5 of these trials were only included in sensitivity analyses as patients on at least one of the treatment arms received additional treatment. This resulted in a subset of 13 trials (3104 patients), which were identified and used for the primary analysis. Within this subset of 13 trials, 1 three‐arm trial combined 2 different forms of CTRT and compared them with a single control arm and 3 four‐arm trials were split into 2 unconfounded comparisons of RT vs CTRT for analysis as separate trials. This resulted in 16 trials included in the primary analysis. In this paper, we will only consider the trials used in the primary analysis and will treat the data in the same way as the original publication.^60^

Across the 3 MAs that form our network of trials, overall survival data were available for 5922 patients from 37 RCTs (35 two‐arm RCTs and 2 three‐arm RCTs). Covariate data were available for stage of disease from 5517 patients from 36 RCTs.

## 3 RESULTS

### 3.1 Application of framework to cervical cancer network

In this section, we illustrate the application of the proposed framework for 1‐stage IPD NMA with TCIs to the cervical cancer network. In this example, we use the 1‐stage IPD Royston‐Parmar NMA model in the Bayesian setting to analyse overall survival.^28^ On the basis of the availability of IPD, stage of disease will be considered for inclusion in a NMA model with TCIs. With a test of linearity, we treat stage of disease as linear throughout the rest of this paper. All models were fitted in WinBUGS^58^ version 1.4.3 and run with 20  000 burn‐in and 20  000 iterations and 2 sets of initial values. Convergence was checked by examining the trace and histograms of the posterior distribution. Models were compared using the DIC statistic.^31^, ^39^ Parameters representing the spline function for the baseline log cumulative hazard function, treatment effects, and inconsistency parameters were fitted with noninformative normal prior distributions. In the RTE model, the treatment effects were modelled using a multivariate normal distribution with the mean coming from a normal distribution and precision from a Wishart distribution. Parameter estimates are presented as LogHR and 95% CrI for the posterior mean. A LogHR of 0 indicates a null effect, and a LogHR less than 0 indicates a beneficial effect relative to the reference treatment, RT.

Step 1: All pairwise treatment comparisons were assessed for evidence of heterogeneity using the Cochran Q statistic and the I ^2^ statistic.^32^, ^33^ There was no evidence of heterogeneity within the RT vs CTRT (P=.625, Table 1) and CT+RT vs CT+S (P=.939) comparisons while there was some evidence of statistical heterogeneity in the RT vs CT+S (P=.065) comparison and substantial heterogeneity in the RT vs CT+RT comparison (P<.001, also noted in the original publication^61^. The baseline characteristics of all trials were compared. A prespecified analysis of RT vs CT+RT identified a difference in treatment effect by chemotherapy cycle length. Therefore, CT+RT was split into 2 treatments on the basis of the length of chemotherapy cycles. Throughout the rest of this paper, trials with chemotherapy cycles less than or equal to 14  days will be referred to as “short cycles” and trials with chemotherapy cycles greater than 14  days will be referred to as “long cycles.” No evidence of heterogeneity was found in the RT vs CT+RT long cycles comparison (P=.263). However, there was evidence of heterogeneity in the RT vs CT+RT short cycles comparison (P=.002). Heterogeneity can also be assessed visually from the forest plots in Figure 2. Treatment effects are presented in Table 1.
  **Table 1:** Cervical cancer meta‐analysis results using Royston‐Parmar models

Comparison	FTE^a^	Cochran Q	Global Non‐PH Test
RT vs CTRT	−0.215	12.71, 15 df,	χ ^2^=0.161, 1 df,
	(−0.336, −0.086)	P=.625	P=.688
RT vs CT+RT short cycles	−0.191	20.69, 6 df,	χ ^2^=2.522, 1 df,
	(−0.375, −0.007)	P=.002	P=.112
RT vs CT+RT long cycles	0.227	12.34, 10 df,	χ ^2^=0.006, 1 df,
	(0.073, 0.385)	P=.263	P=.944
RT vs CT+S	−0.447	8.85, 4 df,	χ ^2^=0.118, 1 df,
	(−0.654, −0.243)	P=.065	P=.731
CT+RT vs CT+S	−0.444	0.01, 1 df,	χ ^2^=0.164, 1 df,
	(−0.830, −0.061)	P=.939	P=.686

Abbreviations: CT+RT, neoadjuvant chemotherapy plus radiotherapy; CT+S, neoadjuvant chemotherapy plus surgery; CTRT, chemoradiation; FTE, fixed treatment effect; PH, proportional hazards; RT, radiotherapy.^a^Values are log hazard ratios and 95% credible intervals.
  **Figure 2:**
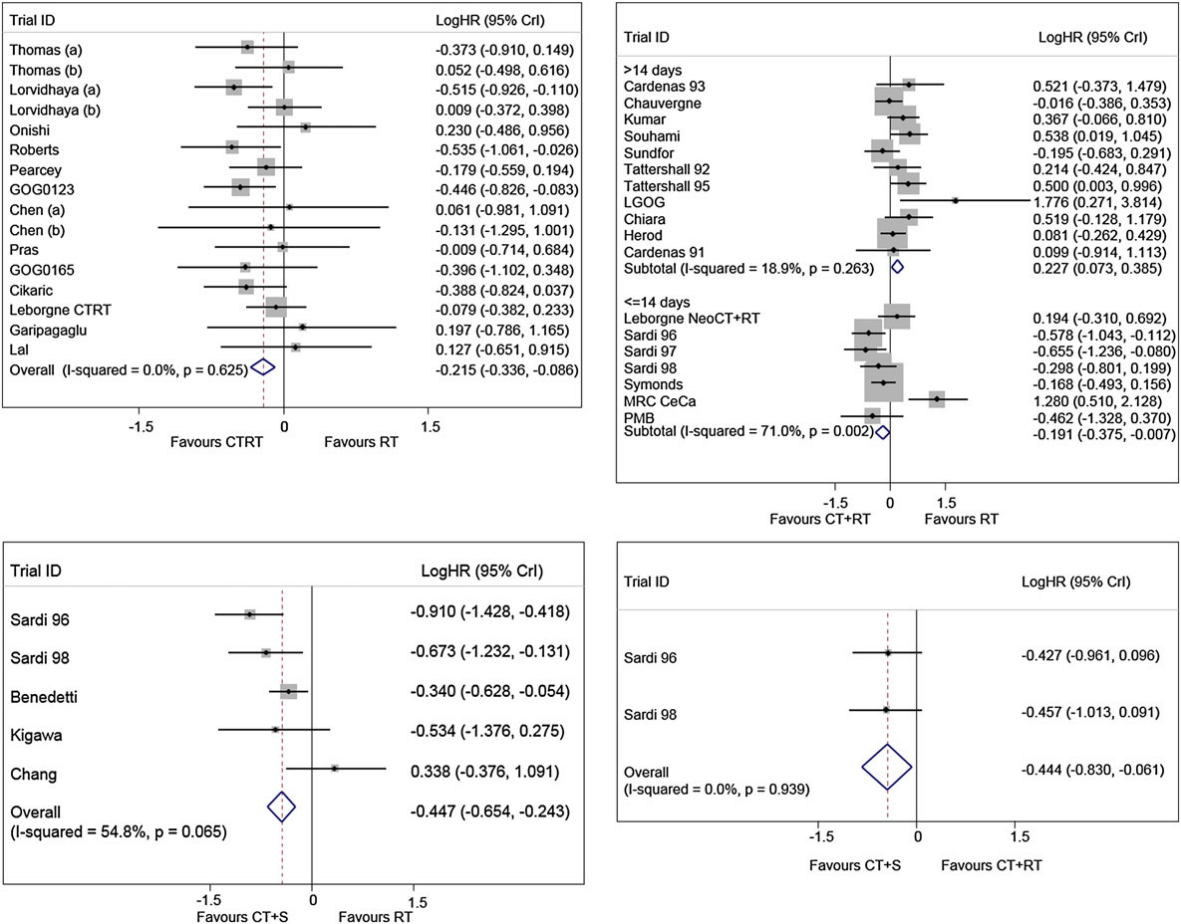
Cervical cancer meta‐analysis plots. Trial results come from a fixed treatment effect Royston‐Parmar model. Overall results come from a 1‐stage IPD fixed treatment effect Royston‐Parmar MA model. Top left: RT vs CTRT. Top right: RT vs CT+RT. Bottom left: RT vs CT+S. Bottom right: CT+RT vs CT+S. CrI indicates credible interval; CT+RT, neoadjuvant chemotherapy plus radiotherapy; CT+S, neoadjuvant chemotherapy plus surgery; CTRT, chemoradiation; LogHR, log hazard ratio; MA, meta‐analysis; RT, radiotherapy
  We also assessed the assumption of proportional hazards (PH). Following the methods described by Freeman and Carpenter,^28^ we performed a global test of nonproportionality, which was not significant for any of the pairwise comparisons; therefore, we continue under the assumption of PH in the cervical cancer network.
Step 2: RT was chosen as the reference treatment because it was the previous standard of care. We included the RT vs CTRT, RT vs CT+RT, and RT vs CT+S treatment contrasts as parameters in the NMA model resulting in the treatment effect for CT+RT vs CT+S being estimated through the consistency equation.
Step 3: A 1‐stage IPD Royston‐Parmar NMA model was fitted to the cervical cancer network using both FTE and RTE with the results presented in Table 2. The DIC provides only weak evidence in favour of the RTE model (FTE DIC  = 12 321.5, RTE DIC  = 12 315.8). However, owing to the presence of heterogeneity in the RT vs CT+S short cycles comparison, identified in step 1, the RTE model was deemed to be the most appropriate model.
  **Table 2:** Posterior mean and 95% credible intervals for treatment effects from NMA models

Treatments	FTE	RTE	RTE + Stage^a^
CTRT	−0.211 (−0.337, −0.087)	−0.207 (−0.374, −0.046)	−0.198 (−0.346, −0.031)
CT+RT short cycles	0.028 (−0.164, 0.220)	0.086 (−0.229, 0.428)	0.005 (−0.320, 0.328)
CT+RT long cycles	0.223 (0.065, 0.380)	0.273 (0.031, 0.538)	0.254 (0.008, 0.540)
CT+S	−0.396 (−0.611, −0.185)	−0.333 (−0.701, 0.011)	−0.372 (−0.803, 0.056)
Stage			0.561 (0.475, 0.641)

Abbreviations: CT+RT, neoadjuvant chemotherapy plus radiotherapy; CT+S, neoadjuvant chemotherapy plus surgery; CTRT, chemoradiation; FTE, fixed treatment effect; NMA, network meta‐analysis; RT, radiotherapy; RTE, random treatment effect.^a^Stage is fitted as a random trial‐level effect.
Step 4: In Figure 3, the direct and indirect treatment effects differ from each other with the network estimates balancing out these 2 sources of information. The direct and indirect treatment effects are estimated through the inclusion of an inconsistency parameter, which was estimated as −0.484 (95% CrI: −1.314, 0.354). The Cochran Q statistic showed some evidence of inconsistency between designs (Q=10.32, 2  df, P=.006). The inconsistency between designs is driven by one trial,^62^ which had a treatment effect estimate more extreme than that of the other trials.
  **Figure 3:**
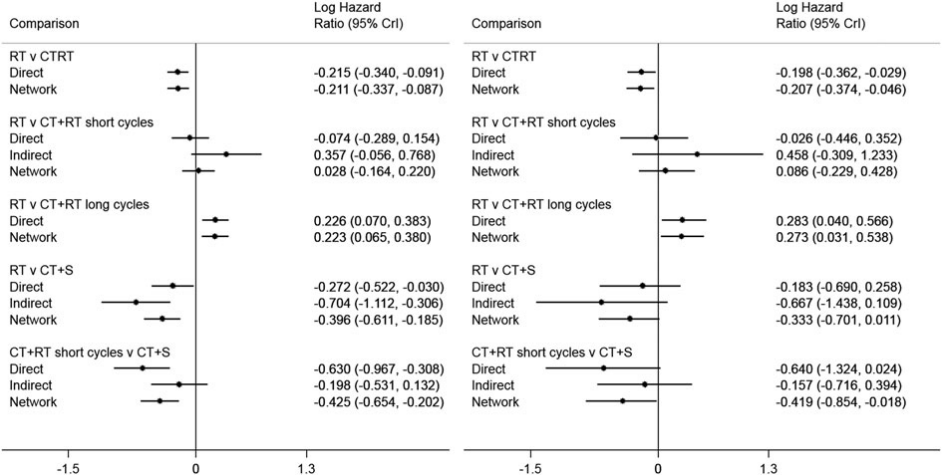
NMA results for the cervical cancer network. Left: Fixed treatment effect. Right: Random treatment effect. CT+RT indicates neoadjuvant chemotherapy plus radiotherapy; CT+S, neoadjuvant chemotherapy plus surgery; CTRT, chemoradiation; NMA, network meta‐analysis; RT, radiotherapy
  In addition, we also assessed globally the assumption of PH across the network using the method recommended by Freeman and Carpenter.^28^ The Wald test for non‐PH from the RTE model with random treatment‐ln(time) interactions gave χ ^2^=0.324 on 3 df(P=.955) giving no evidence of non‐PH within the network.
Step 5: A linear effect of stage of disease was assumed, which could take the values 0 = stages IA to IIA, 1 = stage IIB, and 2 = stages IIIA to IVA. We assessed this assumption by conducting a Wald test for each trial, which included patients covering all 3 categories of stage of disease. As each trial is independent, we summed together the chi‐squared statistics to provide an overall test of the linearity assumption. This gave χ ^2^=8.19 on 12 df and P=.77. Therefore, we proceeded with the assumption of a linear effect for stage of disease.
  Thirteen trials had at least one patient with missing stage data. One of these trials had missing stage data for all patients. To impute values of missing stage of disease (whether explicitly, using multiple imputation, or as part of a Bayesian model), we need to assume a distribution. We used a truncated normal distribution, which we believe is a reasonable approximation for a clinical severity measure of this kind. This is especially so as (in common with other settings) the majority of information is recovered by bringing the observed data on patients with missing stage into the model, and not on the stage coefficients themselves. In WinBUGS, the normal distribution was truncated through the use of the “I” function to restrict missing covariates to take values between 0 and 2. Results with a categorical stage model were similar, but we found it harder to obtain convergence. 
Step 6: A common effect of stage of disease appeared to be inappropriate as the distribution of stage of disease varies across trials and the network includes trials of varying sizes. In addition, the DIC showed that a random effect of stage was most appropriate. Therefore, an RTE model with random trial‐level effect of stage of disease was fitted.
Step 7: As expected, when included as a covariate, the parameter estimate for stage of disease suggests that overall survival is reduced as stage of disease increases (LogHR  =  0.561, 95% CrI: 0.475, 0.641; Table 2). Despite the inclusion of stage of disease as a covariate, the treatment effect for CTRT compared with RT remained statistically significant.
Step 8: A RTE model with fixed trial‐level effect of stage of disease and random trial‐level effect of treatment‐stage interactions separating out the within‐ and across‐trial interactions was fitted. There were no statistically significant interactions between treatment and stage of disease (Table 3). The CrI for the RT vs CT+S comparison are much wider, relative to the other treatment comparisons, possibly reflecting the small amount of within‐trial interaction. In this comparison, there are only 2 trials that have patients distributed over more than one value of stage and can therefore contribute to the within‐trial interaction.
  **Table 3:** Posterior mean and 95% credible intervals for treatment and treatment‐stage interaction effects from NMA models including treatment‐stage interactions with within‐ and across‐trial interactions separated and combined^a^

	Within‐ and Across‐trial	Within‐ and Across‐trial
Treatments	Interactions Separated	Interactions Combined
RT vs CTRT	−0.421 (−0.910, 0.101)	−0.428 (−0.738, −0.114)
RT vs CT+RT short cycles	−0.007 (−0.519, 0.550)	0.118 (−0.273, 0.596)
RT vs CT+RT long cycles	0.100 (−0.551, 0.670)	0.099 (−0.426, 0.613)
RT vs CT+S	0.332 (−0.593, 1.102)	−0.195 (−0.855, 0.380)
RT vs CTRT—stage within	0.176 (−0.069, 0.417)	
RT vs CT+RT—stage within	−0.035 (−0.285, 0.204)	
RT vs CT+S—stage within	0.172 (−0.459, 0.776)	
RT vs CTRT—stage across	0.165 (−0.279, 0.584)	
RT vs CT+RT—stage across	0.110 (−0.315, 0.545)	
RT vs CT+S—stage across	−0.563 (−1.319, 0.230)	
RT vs CTRT—stage combined		0.170 (−0.043, 0.373)
RT vs CT+RT—stage combined		0.006 (−0.234, 0.212)
RT vs CT+S—stage combined		−0.120 (−0.635, 0.415)

Abbreviations: CT+RT, neodadjuavnt chemotherapy plus radiotherapy; CT+S, neoadjuvant chemotherapy plus surgery; NMA, network meta‐analysis; RT, radiotherapy.^a^Reference level is stages IA to IIA.
  A visual assessment of the consistency of the within‐ and across‐trial interactions was conducted by plotting the parameter estimates for the treatment‐stage interactions (Figure 4). To determine whether any information was gained from the NMA, we also plotted the MA estimates from a FTE model (Figure 4). We used a relatively strict criterion considering agreement to be shown if the within‐trial interaction was within half a standard error of the across‐trial interaction. For the RT vs CTRT comparison, there is agreement between the within‐ and across‐trial interactions. This is in line with our expectations as the RT vs CTRT comparison was a branch of the network without any indirect evidence informing the comparison. However, in the case of RT vs CT+RT and RT vs CT+S, the within‐ and across‐trial interactions do not agree. For example, the LogHR of the within‐trial interaction for CT+RT is −0.035 (95% CrI: −0.285, 0.204) and the LogHR for the across‐trial interaction is 0.110 (95% CrI: −0.315, 0.545; Table 3). The within‐ and across‐trial interactions are not consistent with each other, and the across‐trial interaction could be subject to ecological bias. Therefore, we should focus on the within‐trial interaction only. For the RT vs CT+RT and RT vs CT+S comparisons, further investigation into the difference between the within‐ and across‐trial interactions may be required as these comparisons could be subject to ecological bias.
  **Figure 4:**
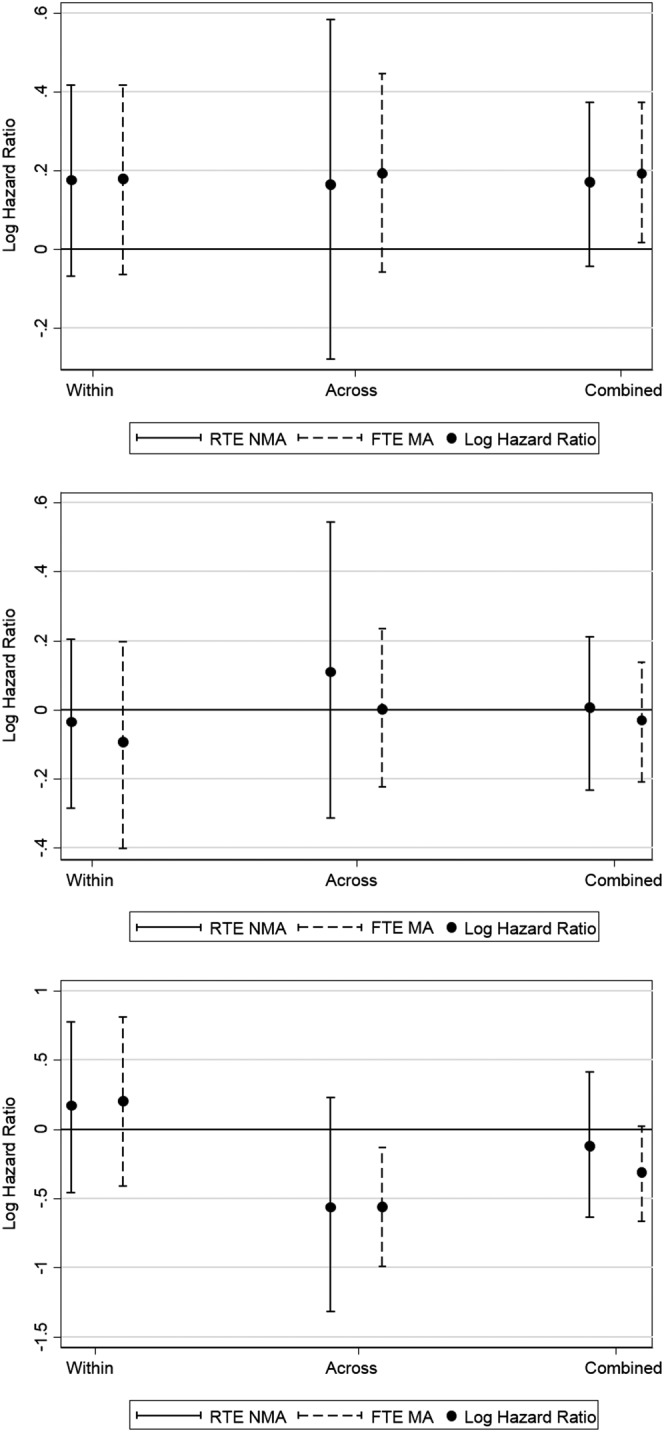
Treatment‐stage interaction parameter estimates. Top: RT vs CTRT. Middle: RT vs CT+RT. Bottom: RT vs CT+S. Solid lines represent NMA estimates. Dashed lines represent pairwise MA estimates. CT+RT indicates neoadjuvant chemotherapy plus radiotherapy; CT+S, neoadjuvant chemotherapy plus surgery; CTRT, chemoradiation; FTE, fixed treatment effect; MA, meta‐analysis; NMA, network meta‐analysis; RT, radiotherapy; RTE, random treatment effect
Step 9: To fully illustrate our framework, we also fitted a model combining the within‐ and across‐trial interactions (Table 3). However, as mentioned in step 8, the within‐ and across‐trial interactions should remain separated. Additionally, a sensitivity analysis in which patients with missing stage of disease were excluded was conducted (Table S2.1). Tables  3 and S2.1 show good agreement between the 2 models. However, in Table S2.1, the across‐trial interaction from the CT+S and stage interaction was statistically significant.

## 4 DISCUSSION

NMA with TCIs has the potential to identify groups of patients most likely to respond to treatment. We have proposed a practical framework that aims to encourage researchers to conduct 1‐stage IPD NMA with TCIs in a systematic and appropriate manner. We have successfully applied the framework to a cervical cancer network. The framework highlights the importance of preliminary analyses to consider issues such as heterogeneity and inconsistency, which may inform decisions around the most appropriate modelling assumptions. This framework is deliberately generic, so that it can be applied to a range of outcomes (eg, binary, continuous, and time‐to‐event) and has the potential to improve the conduct and analysis of NMA with TCIs.

In the cervical cancer network, we showed that stage of disease had a statistically significant effect on overall survival with advanced disease increasing the risk of death. Owing to the presence of heterogeneity in the network, a RTE model was considered to be the most appropriate. There was no evidence of a treatment‐stage interaction on the basis of the within‐trial interaction for any of the treatments leading to the conclusion that stage of disease did not modify the treatment effect. On the basis of our relatively strict criterion, the treatment‐stage interaction models showed a difference between the within‐ and across‐trial interactions for some of the comparisons, and it was therefore most appropriate to separate out the within‐ and across‐trial interactions. The small difference in the NMA and MA estimates of the across‐trial interaction suggested that some across‐trial interactions might have been gained from the network. Our criterion for assessing agreement between the within‐ and across‐trial interactions is arguably somewhat strict and arbitrary. However, we feel it is better to be cautious as a number of TCIs identified in the literature have subsequently been debunked.^6^

The cervical cancer network is a small, well‐connected network with a lot of direct evidence. Despite this, we were still able to show that some across‐trial interaction is gained when conducting a NMA. Information (in the statistical sense of the inverse of the squared standard error) is gained in a NMA when the direct evidence and indirect evidence are consistent. In practice, not all networks will contain as much direct evidence as the cervical cancer network. Therefore, we would expect NMA to contribute a greater amount of statistical information on an across‐trial interaction in a consistent network where some treatment comparisons are only informed by a small amount of direct evidence. We may also expect to gain more information when using a fixed‐effects NMA.

A reviewer suggested that (as the IPD data are available) the following simpler analysis may be preferable: (1) perform a 2‐stage MA of the within‐study interaction effects and then (2) derive the interaction effects within each study using the IPD and then perform NMA on these. In applications, this could provide a useful cross‐check of the results. However, in our setting, the network gives us the best treatment estimates against which to estimate an interaction. Further, NMA goes beyond just estimating treatment and interaction effects: A key motivation for NMA is to rank treatments in terms of efficacy. The inclusion of TCIs allows us to consider whether the ranking of treatments varies by covariate level. Indeed, if a TCI is present, we should rank treatments for each level of the covariate.

With the cervical cancer network, we did not need to include an inconsistency parameter. However, in a network with one treatment loop, if an inconsistency parameter is included, then it may also be appropriate to allow for inconsistency in the TCIs. This would be equivalent to conducting separate pairwise MA with TCI, and nothing would be gained from conducting a NMA. We also only considered one covariate, whereas in practice, researchers may wish to consider multiple covariates. In this case, we would recommend considering each covariate on its own initially before combining any covariates identified as being clinically important within a NMA model.

By definition, NMA uses both the within‐ and across‐trial interactions. Using the across‐trial interaction requires the assumption of no unmeasured confounding, but unfortunately, this assumption will always be hard to test. Making this assumption allows information to be gained from the network to inform both treatment effect estimates and TCIs. Each trial contributes to the within‐trial interaction, which is estimated using patient‐level covariates. Meanwhile, the across‐trial interaction is estimated through the relation of the trial‐level aggregated covariates.^13^ Although combining within‐ and across‐trial interactions can result in greater power to detect TCIs, the across‐trial interaction can introduce ecological bias.^3^ It is therefore important that the within‐ and across‐trial interactions for TCIs can be separated out.^6^ Separating out the within‐ and across‐trial interactions allows the influence of the across‐trial interaction on the TCI to be assessed and allows researchers to identify which data source is driving the TCI.

## 5 CONCLUSION

NMA with TCIs has the potential to identify groups of patients most likely to respond to treatment. To do this, NMA requires the use of both within‐ and across‐trial interactions. However, the across‐trial interaction can be subject to ecological bias. Therefore, it is important that the within‐ and across‐trial interactions in a NMA can be separated and checked for agreement. We have shown that NMA models can be parameterised to separate out the within‐ and across‐trial interactions. Our proposed framework incorporates the separation of within‐ and across‐trial interactions, can be applied to any outcome, outlines the steps to conducting NMA with TCIs in a systematic manner, provides practical guidance for researchers, and reduces the risk of unduly optimistic interpretation of TCIs.

## HIGHLIGHTS

What is already known?

- Treatment‐covariate interactions explore how a treatment effect varies in relation to a patient‐level covariate.
- Treatment‐covariate interactions contain within‐ and across‐trial interactions, where the across‐trial interaction is susceptible to confounding and ecological bias.

What is new?

- We propose a 9‐step framework for IPD NMA with TCIs. 
- We use a cervical cancer example to show how to implement the framework, parameterise the NMA models to separate out the within‐ and across‐trial interactions, and assess the consistency of the within‐ and across‐trial interactions.

Potential impact for  *Research Synthesis Methods*  readers outside the authors' field: 

- This framework provides practical guidance for researchers outlining the steps for conducting NMA with TCIs in a systematic manner, reducing the risk of overly optimistic interpretation of TCIs.

## Supporting information

SUPPORTING INFORMATION

Table B.1: Posterior mean and 95% credible intervals for treatment and treatment‐stage interaction effects from NMA models including treatment‐stage interactions with within and across trial interactions separated and combined. Patients with missing stage of disease are excluded. Reference level is stages 1A‐2A. RT = radiotherapy, CT+RT = neodadjuavnt chemotherapy plus radiotherapy, CT+S= neoadjuvant chemotherapy plus surgery.
