# Supplementary material for: A framework for identifying treatment‐covariate interactions in individual participant data network meta‐analysis
Source: Res Synth Methods. 2018 Jun 11;9(3):393–407. doi: 10.1002/jrsm.1300 (PMC6159880; doi:10.1002/jrsm.1300)
Supplement: Supplementary file 1 — Table B.1: Posterior mean and 95% credible intervals for treatment and treatment‐stage interaction effects from NMA models including treatment‐stage interactions with within and across trial interactions separated and combined. Patients with missing stage of disease are excluded. Reference level is stages 1A‐2A. RT = radiotherapy, CT+RT = neodadjuavnt chemotherapy plus radiotherapy, CT+S= neoadjuvant chemotherapy plus surgery. [file JRSM-9-393-s001.pdf]

A framework for identifying treatment-covariate interactions in individual participant data network meta-analysis: Supplementary material

S. C. Freeman, D. Fisher, J. F. Tierney, J. R. Carpenter

A Additional parameterisation of within and across trial interactions

The random treatment effect model with random trial-level treatment-covariate interaction and random trial-level effect of the patient-level covariate, z_{ij}, which separates the within and across trial interactions is:

$$\begin{aligned}
\ln\{H_j(t|x_{ij})\} &= s_j\big(\ln(t)\big) + \beta_{1j}\text{trt1}_{ij} + \cdots + \beta_{qj}\text{trtq}_{ij} + \alpha_j(z_{ij} - \bar{z}_j) \\
&+ \delta_{A1j}\text{trt1}_{ij}(z_{ij} - \bar{z}_j) + \cdots + \delta_{Aqj}\text{trtq}_{ij}(z_{ij} - \bar{z}_j) \\
&+ \delta_{B1j}\text{trt1}_{ij}\bar{z_j} + \cdots + \delta_{Bqj}\text{trtq}_{ij}\bar{z_j} \\
\beta_j &\sim MVN(\mu_\beta, T_\beta) \\
\alpha_j &\sim N(\theta, \sigma^2) \\
\delta_{Aj} &\sim MVN(\mu_{\delta_A}, T_{\delta_A}) \\
\delta_{Bj} &\sim MVN(\mu_{\delta_B}, T_{\delta_B})
\end{aligned}$$

where z_{ij} is the covariate value for patient i in trial j with coefficient α_j for trial j and $\bar{z}_j$ is the mean value of z_{ij} for trial j. In this model $\beta_{1j}, \ldots, \beta_{qj}$ are random treatment effects. The within trial interaction is estimated by μ_{δ_A} whilst the across trial information is estimated by $\mu_{\delta_A} + \mu_{\delta_B}$.

The random treatment effect model with random trial-level treatment-covariate interaction and random trial-level effect of the patient-level covariate, z_{ij}, which combines the within and across trial interactions is:

$$\begin{aligned}
\ln\{H_j(t|x_{ij})\} &= s_j\big(\ln(t)\big) + \beta_{1j}\text{trt1}_{ij} + \cdots + \beta_{qj}\text{trtq}_{ij} + \alpha_j z_{ij} \\
&+ \delta_{1j}\text{trt1}_{ij}z_{ij} + \cdots + \delta_{qj}\text{trtq}_{ij}z_{ij} \\
\beta_j &\sim MVN(\mu_\beta, T_\beta) \\
\delta_j &\sim MVN(\mu_\delta, T_\delta) \\
\alpha_j &\sim N(\theta, \sigma^2)
\end{aligned}$$

where z_{ij} is the covariate value for patient i in trial j with coefficient α_j for trial j, $\beta_{1j}, \ldots, \beta_{qj}$ are random treatment effects and $\delta_{1j}, \ldots, \delta_{qj}$ are random treatment-covariate interaction effects.

B Sensitivity analysis: Cervical cancer NMA with treatment-stage interactions excluding patients with missing data

Table B.1: Posterior mean and 95% credible intervals for treatment and treatment-stage interaction effects from NMA models including treatment-stage interactions with within and across trial interactions separated and combined. Patients with missing stage of disease are excluded. Reference level is stages 1A-2A. RT = radiotherapy, CT+RT = neodadjuavnt chemotherapy plus radiotherapy, CT+S = neoadjuvant chemotherapy plus surgery.

	**Within & across trial interactions separated**	**Within & across trial interactions combined**
RT v CTRT	−0.460 (−1.121, 0.123)	−0.462 (−0.802, −0.128)
RT v CT+RT short cycles	−0.086 (−0.702, 0.610)	0.067 (−0.396, 0.575)
RT v CT+RT long cycles	0.043 (−0.623, 0.745)	0.181 (−0.440, 0.735)
RT v CT+S	0.303 (−0.537, 1.245)	−0.020 (−0.780, 0.671)
CTRT - stage within	0.146 (−0.087, 0.403)	
CT+RT - stage within	−0.063 (−0.317, 0.187)	
CT+S - stage within	−0.213 (−0.811, 0.374)	
CTRT - stage across	0.189 (−0.260, 0.718)	
CT+RT - stage across	0.219 (−0.281, 0.708)	
CT+S - stage across	−0.461 (−1.338, 0.372)	
CTRT - stage combined		0.178 (−0.040, 0.400)
CT+RT - stage combined		0.023 (−0.218, 0.260)
CT+S - stage combined		−0.251 (−0.723, 0.302)
